# Prevalence of congenital coronary artery abnormalities in south asian patients with pulmonary stenosis and VSD

**DOI:** 10.1186/1532-429X-17-S1-P213

**Published:** 2015-02-03

**Authors:** Vimal Raj, Vinay Belaval, Manoj KV, Karthik GA

**Affiliations:** Imaging, Narayana Hrudayalaya Hospitals, Bangalore, India

## Background

Incidence of anomalous coronary anatomy (ACA) in caucasian and middle eastern patients with ventricular septal defect (VSD) and pulmonary stenosis (PS) is between 2-9%. Prevalence of ACA in South Asian (SA) patients is unknown. Patients with VSD and PS may have Tetrology of Fallot (TOF) or Double Outlet Right ventricle (DORV) morphology. This study looks at the prevalence of ACA in SA patients with TOF/DORV. Pre-operative assessment of ACA is of paramount importance in these patients as it alters surgical procedure.

## Methods

Retrospective analysis of all Multi Detector Computed Tomography (MDCT) and Cardiac Magnetic Resonance (CMR) examinations of SA patients with TOF/DORV was performed in our institute over 4 years (June 2011 to June 2014). Coronary anomalies were classified into Variant Coronary Anatomy (VCA) and ACA. VCA included coronaries that coursed close to the right ventricular outflow tract (RVOT) while ACA included anomalous origin or branching of coronaries.

## Results

A total of 1011 scans were performed during this period. Of these, 95 were excluded due to suboptimal visualisation of the coronary arteries. Coronary anomalies were seen in 146 (16%) patients. Most common abnormality was VCA, seen in 78 (8.5%) patients with large conus or acute marginal or septal branch crossing the RVOT. ACA was seen in 68 patients (7.4%), with left anterior descending (LAD)/ Left main stem (LMS) arising from right coronary sinus in 35 patients (4%) and right coronary artery (RCA) arising from LAD/LMS in 16 patients (1.7%). OTher anomalies included left coronary artery origin from pulmonary artery (ALCAPA), coronary cameral fistula, single coronary arteries, dual LAD and conus branch from coronary sinus.

## Conclusions

Coronary artery anomalies are common in SA patients with VSD and PS. Optimal imaging is therefore essential to establish coronary anatomy prior to surgery.

**Figure 1:**
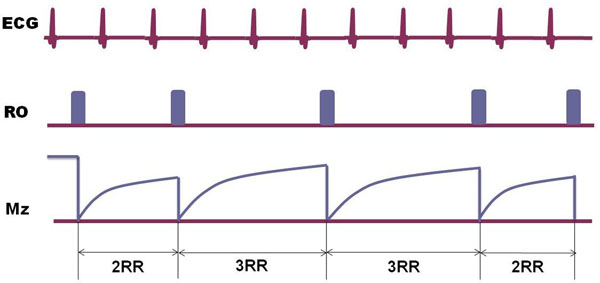
Figure 1

